# 1,2-Bis[*N*′-(2,2-dimethyl­propionyl)thio­ureido]cyclo­hexa­ne

**DOI:** 10.1107/S1600536808011495

**Published:** 2008-04-30

**Authors:** M. Sukeri M. Yusof, Noor Adila C. Ayob, Maisara A. Kadir, Bohari M. Yamin

**Affiliations:** a Department of Chemical Sciences, Faculty of Science and Technology, Universiti Malaysia Terengganu, Mengabang Telipot, 21030 Kuala Terengganu, Malaysia; b School of Chemical Sciences and Food Technology, Universiti Kebangsaan Malaysia, 43600 Bangi, Selangor, Malaysia

## Abstract

In the title compound, C_18_H_32_N_4_O_2_S_2_, the dihedral angle between the two thio­urea groups is 78.55 (7)°. The mol­ecular conformation is stabilized by intra­molecular N—H⋯O hydrogen bonds and the crystal structure is stabilized by inter­molecular N—H⋯O and C—H⋯O hydrogen bonds, forming centrosymmetric dimers.

## Related literature

For related crystal structures, see: Thiam *et al.* (2008 ▶). For bond-length data, see: Allen *et al.* (1987 ▶).
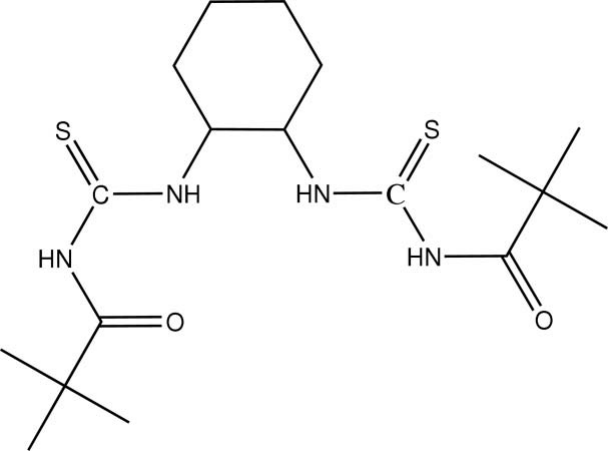


## Experimental

### 

#### Crystal data

C_18_H_32_N_4_O_2_S_2_
*M* *_r_* = 400.60
Monoclinic, 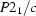

*a* = 10.960 (2) Å
*b* = 19.065 (4) Å
*c* = 10.378 (2) Å
β = 96.112 (4)°
*V* = 2156.1 (8) Å^3^
*Z* = 4
Mo *K*α radiation
μ = 0.27 mm^−1^
*T* = 298 (2) K
0.48 × 0.41 × 0.37 mm

#### Data collection

Bruker SMART APEX CCD area-detector diffractometer
Absorption correction: multi-scan (*SADABS*; Bruker, 2000 ▶) *T*_min_ = 0.883, *T*_max_ = 0.908
10928 measured reflections
3779 independent reflections
2828 reflections with *I* > 2σ(*I*)
*R*_int_ = 0.026

#### Refinement

*R*[*F* ^2^ > 2σ(*F* ^2^)] = 0.044
*wR*(*F* ^2^) = 0.124
*S* = 1.07
3779 reflections
241 parameters
H-atom parameters constrained
Δρ_max_ = 0.30 e Å^−3^
Δρ_min_ = −0.22 e Å^−3^

### 

Data collection: *SMART* (Bruker, 2000 ▶); cell refinement: *SAINT* (Bruker, 2000 ▶); data reduction: *SAINT*; program(s) used to solve structure: *SHELXS97* (Sheldrick, 2008 ▶); program(s) used to refine structure: *SHELXL97* (Sheldrick, 2008 ▶); molecular graphics: *SHELXTL* (Sheldrick, 2008 ▶); software used to prepare material for publication: *SHELXTL*, *PARST* (Nardelli, 1995 ▶) and *PLATON* (Spek, 2003 ▶).

## Supplementary Material

Crystal structure: contains datablocks global, I. DOI: 10.1107/S1600536808011495/bt2701sup1.cif

Structure factors: contains datablocks I. DOI: 10.1107/S1600536808011495/bt2701Isup2.hkl

Additional supplementary materials:  crystallographic information; 3D view; checkCIF report

## Figures and Tables

**Table 1:** Hydrogen-bond geometry (Å, °)

*D*—H⋯*A*	*D*—H	H⋯*A*	*D*⋯*A*	*D*—H⋯*A*
N2—H2⋯O1	0.86	1.97	2.650 (2)	135
N3—H3⋯O2	0.86	1.99	2.670 (2)	135
N1—H1⋯O2^i^	0.86	2.25	3.078 (2)	161
C1—H1*B*⋯O2^i^	0.96	2.54	3.462 (3)	161

Symmetry code: (i) 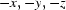.

**Fig. 1.**
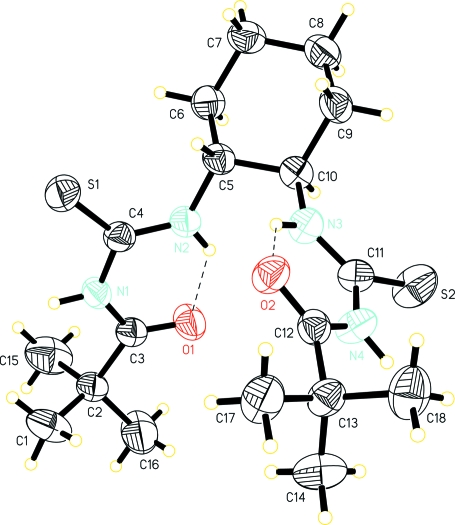
: The molecular structure of the title compound showing the atom-labelling scheme. Displacement ellipsoids are drawn at the 50% probability level. Dashed lines indicate hydrogen bonds.

**Fig. 2.**
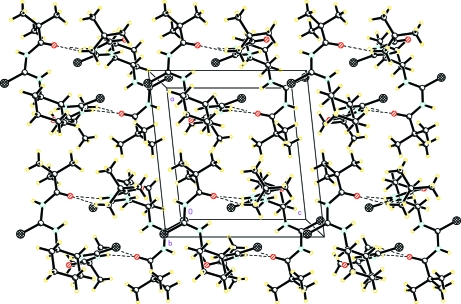
: Packing diagram of the title compound viewed down the b axis. The dashed lines denote hydrogen bonds.

## supplementary crystallographic information

### Comment

The title compound, (I), is a propionylthiourea derivatives and analogous to 1,2-bis(*N*'-benzoylthioureido)benzene, (Thiam *et al.*, 2008), except that the benzene and benzoyl groups are replaced by cyclohexane and 2,2-dimethylpropionyl group respectively (Fig. 1). The bond lengths and angles are in normal ranges (Allen *et al.*, 1987). The   thiourea fragments [S1/N1/N2/C4 and S2/N3/N4/C11] are all planar, with a maximum deviation of 0.024 (2)Å from least-sqares plane for atom N2 and the dihedral angles between them is 78.55 (7)°.

There are two intramolecular hydrogen bonds, N2—H2···O1 and N3—H3···O2 (Table 1), forming two pseudo-six-membered rings (O1···H2—N2—C4—N1—C3—O1 and O2···H3—N3—C11—N4—C12—O2). In the crystal structure, the molecules are linked by intermolecular interactions, N—H···O and C—H···O   forming centrosymmetric dimers  (Fig. 2).

### Experimental

To a stirring acetone solution (75 ml) of pivaloyl chloride (5.0 g, 0.04 mol) and ammonium thiocyanate (3.15 g, 0.04 mol), 1,2-diaminocyclohexane (2.37 g, 0.02 mol) in 40 ml of acetone was added dropwise. The solution mixture was refluxed for 1 h. The resulting solution was poured into a beaker containing some ice blocks. The white precipitate was filtered off and washed with distilled water and cold ethanol before dried under vacuum. Good quality crystals were obtained by recrystallization from DMSO.

### Refinement

After their location in the difference map, all H-atoms were fixed geometrically at ideal positions and allowed to ride on the parent C or N atoms with C—H = 0.93–0.97Å and N—H = 0.86Å with *U*_iso_(H)= 1.2 (CH_2_ and NH) or 1.5*U*_eq_(C)(CH_3_). The methyl groups were allowed to rotate but not to tip.

### Crystal data

**Table:**

C_18_H_32_N_4_O_2_S_2_	*F*_000_ = 864
*M**_r_* = 400.60	*D*_x_ = 1.234 Mg m^−^^3^
Monoclinic, *P*2_1_/*c*	Mo *K*α radiation λ = 0.71073 Å
Hall symbol: -P 2ybc	Cell parameters from 982 reflections
*a* = 10.960 (2) Å	θ = 1.9–25.0º
*b* = 19.065 (4) Å	µ = 0.27 mm^−^^1^
*c* = 10.378 (2) Å	*T* = 298 (2) K
β = 96.112 (4)º	Block, colourless
*V* = 2156.1 (8) Å^3^	0.48 × 0.41 × 0.37 mm
*Z* = 4	

### Data collection

**Table:**

Bruker SMART APEX CCD area-detector diffractometer	3779 independent reflections
Radiation source: fine-focus sealed tube	2828 reflections with *I* > 2σ(*I*)
Monochromator: graphite	*R*_int_ = 0.026
Detector resolution: 83.66 pixels mm^-1^	θ_max_ = 25.0º
*T* = 298(2) K	θ_min_ = 1.9º
ω scans	*h* = −9→13
Absorption correction: multi-scan(SADABS; Bruker, 2000)	*k* = −22→22
*T*_min_ = 0.883, *T*_max_ = 0.908	*l* = −12→11
10928 measured reflections	

### Refinement

**Table:**

Refinement on *F*^2^	Secondary atom site location: difference Fourier map
Least-squares matrix: full	Hydrogen site location: inferred from neighbouring sites
*R*[*F*^2^ > 2σ(*F*^2^)] = 0.044	H-atom parameters constrained
*wR*(*F*^2^) = 0.124	*w* = 1/[σ^2^(*F*_o_^2^) + (0.0662*P*)^2^ + 0.3145*P*] where *P* = (*F*_o_^2^ + 2*F*_c_^2^)/3
*S* = 1.07	(Δ/σ)_max_ = 0.001
3779 reflections	Δρ_max_ = 0.30 e Å^−^^3^
241 parameters	Δρ_min_ = −0.22 e Å^−^^3^
Primary atom site location: structure-invariant direct methods	Extinction correction: none

### Special details

**Table:**

Geometry. All e.s.d.'s (except the e.s.d. in the dihedral angle between two l.s. planes) are estimated using the full covariance matrix. The cell e.s.d.'s are taken into account individually in the estimation of e.s.d.'s in distances, angles and torsion angles; correlations between e.s.d.'s in cell parameters are only used when they are defined by crystal symmetry. An approximate (isotropic) treatment of cell e.s.d.'s is used for estimating e.s.d.'s involving l.s. planes.
Refinement. Refinement of *F*^2^ against ALL reflections. The weighted *R*-factor *wR* and goodness of fit *S* are based on *F*^2^, conventional *R*-factors *R* are based on *F*, with *F* set to zero for negative *F*^2^. The threshold expression of *F*^2^ > σ(*F*^2^) is used only for calculating *R*-factors(gt) *etc*. and is not relevant to the choice of reflections for refinement. *R*-factors based on *F*^2^ are statistically about twice as large as those based on *F*, and *R*- factors based on ALL data will be even larger.

### Fractional atomic coordinates and isotropic or equivalent isotropic displacement parameters (Å^2^)

**Table:**

	*x*	*y*	*z*	*U*_iso_*/*U*_eq_	
S1	0.12804 (6)	0.05301 (3)	−0.07986 (6)	0.0544 (2)	
S2	0.03271 (6)	0.10826 (4)	0.56450 (6)	0.0689 (2)	
O1	0.26724 (16)	−0.05371 (8)	0.29928 (15)	0.0566 (4)	
O2	−0.21983 (13)	0.10785 (8)	0.19001 (14)	0.0464 (4)	
N1	0.20717 (16)	−0.04742 (8)	0.08323 (17)	0.0424 (4)	
H1	0.1996	−0.0721	0.0135	0.051*	
N2	0.18658 (16)	0.06134 (8)	0.17475 (17)	0.0426 (4)	
H2	0.2023	0.0397	0.2473	0.051*	
N3	0.00442 (15)	0.13012 (9)	0.31031 (18)	0.0435 (4)	
H3	−0.0426	0.1273	0.2387	0.052*	
N4	−0.15887 (16)	0.08040 (10)	0.39865 (18)	0.0481 (5)	
H4	−0.1840	0.0617	0.4664	0.058*	
C1	0.1676 (3)	−0.19515 (13)	0.0914 (3)	0.0723 (8)	
H1A	0.1771	−0.2452	0.0940	0.108*	
H1B	0.1685	−0.1790	0.0039	0.108*	
H1C	0.0910	−0.1827	0.1222	0.108*	
C2	0.2729 (2)	−0.16111 (11)	0.1774 (2)	0.0468 (5)	
C3	0.24848 (19)	−0.08288 (11)	0.1939 (2)	0.0415 (5)	
C4	0.17546 (18)	0.02355 (11)	0.0674 (2)	0.0396 (5)	
C5	0.17396 (18)	0.13730 (10)	0.1781 (2)	0.0391 (5)	
H5	0.1138	0.1516	0.1063	0.047*	
C6	0.2965 (2)	0.17181 (11)	0.1603 (2)	0.0462 (5)	
H6A	0.3591	0.1529	0.2239	0.055*	
H6B	0.3190	0.1603	0.0749	0.055*	
C7	0.2932 (2)	0.25092 (11)	0.1746 (2)	0.0523 (6)	
H7A	0.3743	0.2701	0.1682	0.063*	
H7B	0.2375	0.2708	0.1053	0.063*	
C8	0.2512 (2)	0.27047 (12)	0.3043 (2)	0.0564 (6)	
H8A	0.3100	0.2534	0.3736	0.068*	
H8B	0.2469	0.3211	0.3115	0.068*	
C9	0.1265 (2)	0.23904 (11)	0.3176 (2)	0.0480 (6)	
H9A	0.0671	0.2586	0.2513	0.058*	
H9B	0.1014	0.2516	0.4014	0.058*	
C10	0.12704 (18)	0.15951 (10)	0.3048 (2)	0.0396 (5)	
H10	0.1819	0.1401	0.3767	0.048*	
C11	−0.03989 (18)	0.10766 (11)	0.4159 (2)	0.0421 (5)	
C12	−0.24141 (18)	0.07907 (11)	0.2897 (2)	0.0387 (5)	
C13	−0.36266 (19)	0.04254 (11)	0.3039 (2)	0.0436 (5)	
C14	−0.3419 (2)	−0.03052 (14)	0.3627 (3)	0.0676 (8)	
H14A	−0.4196	−0.0533	0.3662	0.101*	
H14B	−0.3006	−0.0265	0.4487	0.101*	
H14C	−0.2926	−0.0577	0.3101	0.101*	
C15	0.3909 (2)	−0.16947 (14)	0.1128 (3)	0.0769 (9)	
H15A	0.4573	−0.1469	0.1647	0.115*	
H15B	0.3805	−0.1484	0.0283	0.115*	
H15C	0.4091	−0.2184	0.1047	0.115*	
C16	0.2847 (4)	−0.19545 (14)	0.3097 (3)	0.1025 (13)	
H16A	0.3535	−0.1757	0.3624	0.154*	
H16B	0.2971	−0.2450	0.3004	0.154*	
H16C	0.2112	−0.1875	0.3502	0.154*	
C17	−0.4349 (2)	0.03560 (15)	0.1708 (2)	0.0607 (7)	
H17A	−0.4482	0.0813	0.1330	0.091*	
H17B	−0.5126	0.0138	0.1795	0.091*	
H17C	−0.3895	0.0073	0.1161	0.091*	
C18	−0.4344 (2)	0.08752 (15)	0.3912 (3)	0.0649 (7)	
H18A	−0.4380	0.1349	0.3598	0.097*	
H18B	−0.3941	0.0866	0.4780	0.097*	
H18C	−0.5161	0.0693	0.3907	0.097*	

### Atomic displacement parameters (Å^2^)

**Table:**

	*U*^11^	*U*^22^	*U*^33^	*U*^12^	*U*^13^	*U*^23^
S1	0.0754 (4)	0.0458 (4)	0.0412 (3)	0.0087 (3)	0.0030 (3)	0.0069 (3)
S2	0.0585 (4)	0.0989 (6)	0.0462 (4)	−0.0190 (4)	−0.0089 (3)	0.0110 (3)
O1	0.0847 (12)	0.0408 (9)	0.0420 (10)	0.0074 (8)	−0.0043 (8)	−0.0010 (7)
O2	0.0466 (9)	0.0556 (9)	0.0366 (9)	−0.0066 (7)	0.0027 (6)	0.0042 (7)
N1	0.0578 (11)	0.0324 (9)	0.0363 (10)	0.0027 (8)	0.0014 (8)	−0.0020 (8)
N2	0.0563 (11)	0.0337 (9)	0.0376 (10)	0.0042 (8)	0.0039 (8)	0.0023 (8)
N3	0.0363 (9)	0.0525 (11)	0.0411 (11)	−0.0040 (8)	0.0011 (8)	−0.0005 (8)
N4	0.0415 (10)	0.0599 (12)	0.0423 (11)	−0.0091 (9)	0.0012 (8)	0.0127 (9)
C1	0.0789 (19)	0.0417 (14)	0.096 (2)	−0.0145 (13)	0.0081 (16)	−0.0041 (14)
C2	0.0633 (15)	0.0318 (11)	0.0454 (13)	0.0014 (10)	0.0067 (11)	0.0026 (10)
C3	0.0472 (12)	0.0355 (11)	0.0416 (13)	−0.0012 (9)	0.0045 (10)	0.0017 (10)
C4	0.0414 (12)	0.0351 (11)	0.0431 (13)	0.0009 (9)	0.0080 (9)	0.0011 (9)
C5	0.0413 (11)	0.0320 (11)	0.0437 (12)	0.0075 (9)	0.0031 (9)	0.0018 (9)
C6	0.0430 (12)	0.0453 (12)	0.0514 (14)	0.0050 (10)	0.0094 (10)	−0.0004 (10)
C7	0.0501 (13)	0.0433 (13)	0.0645 (16)	−0.0060 (10)	0.0108 (11)	0.0003 (11)
C8	0.0619 (15)	0.0435 (13)	0.0646 (16)	−0.0077 (11)	0.0103 (12)	−0.0097 (12)
C9	0.0486 (13)	0.0422 (12)	0.0543 (14)	0.0061 (10)	0.0105 (11)	−0.0072 (11)
C10	0.0326 (11)	0.0402 (12)	0.0456 (13)	0.0005 (9)	0.0019 (9)	−0.0014 (10)
C11	0.0402 (12)	0.0401 (12)	0.0456 (13)	−0.0009 (9)	0.0025 (10)	0.0032 (10)
C12	0.0417 (12)	0.0366 (11)	0.0377 (12)	0.0042 (9)	0.0044 (9)	−0.0012 (9)
C13	0.0362 (11)	0.0522 (13)	0.0426 (13)	−0.0029 (10)	0.0058 (9)	0.0025 (10)
C14	0.0560 (15)	0.0608 (16)	0.086 (2)	−0.0131 (13)	0.0092 (14)	0.0163 (15)
C15	0.0644 (17)	0.0511 (15)	0.117 (3)	0.0140 (13)	0.0166 (17)	0.0041 (16)
C16	0.214 (4)	0.0405 (15)	0.0529 (18)	0.0181 (19)	0.011 (2)	0.0113 (13)
C17	0.0458 (13)	0.0868 (18)	0.0492 (15)	−0.0151 (13)	0.0037 (11)	−0.0059 (13)
C18	0.0494 (14)	0.088 (2)	0.0584 (17)	0.0067 (13)	0.0092 (12)	−0.0083 (14)

### Geometric parameters (Å, °)

**Table:**

S1—C4	1.659 (2)	C7—C8	1.515 (3)
S2—C11	1.658 (2)	C7—H7A	0.9700
O1—C3	1.224 (2)	C7—H7B	0.9700
O2—C12	1.217 (2)	C8—C9	1.512 (3)
N1—C3	1.368 (3)	C8—H8A	0.9700
N1—C4	1.402 (3)	C8—H8B	0.9700
N1—H1	0.8600	C9—C10	1.522 (3)
N2—C4	1.322 (3)	C9—H9A	0.9700
N2—C5	1.455 (2)	C9—H9B	0.9700
N2—H2	0.8600	C10—H10	0.9800
N3—C11	1.317 (3)	C12—C13	1.521 (3)
N3—C10	1.463 (3)	C13—C17	1.522 (3)
N3—H3	0.8600	C13—C18	1.525 (3)
N4—C12	1.371 (3)	C13—C14	1.528 (3)
N4—C11	1.397 (3)	C14—H14A	0.9600
N4—H4	0.8600	C14—H14B	0.9600
C1—C2	1.526 (4)	C14—H14C	0.9600
C1—H1A	0.9600	C15—H15A	0.9600
C1—H1B	0.9600	C15—H15B	0.9600
C1—H1C	0.9600	C15—H15C	0.9600
C2—C16	1.514 (3)	C16—H16A	0.9600
C2—C15	1.528 (4)	C16—H16B	0.9600
C2—C3	1.528 (3)	C16—H16C	0.9600
C5—C10	1.522 (3)	C17—H17A	0.9600
C5—C6	1.524 (3)	C17—H17B	0.9600
C5—H5	0.9800	C17—H17C	0.9600
C6—C7	1.516 (3)	C18—H18A	0.9600
C6—H6A	0.9700	C18—H18B	0.9600
C6—H6B	0.9700	C18—H18C	0.9600
			
C3—N1—C4	129.13 (18)	C8—C9—C10	112.04 (18)
C3—N1—H1	115.4	C8—C9—H9A	109.2
C4—N1—H1	115.4	C10—C9—H9A	109.2
C4—N2—C5	124.21 (18)	C8—C9—H9B	109.2
C4—N2—H2	117.9	C10—C9—H9B	109.2
C5—N2—H2	117.9	H9A—C9—H9B	107.9
C11—N3—C10	125.39 (18)	N3—C10—C5	108.89 (16)
C11—N3—H3	117.3	N3—C10—C9	111.49 (16)
C10—N3—H3	117.3	C5—C10—C9	110.94 (17)
C12—N4—C11	129.61 (18)	N3—C10—H10	108.5
C12—N4—H4	115.2	C5—C10—H10	108.5
C11—N4—H4	115.2	C9—C10—H10	108.5
C2—C1—H1A	109.5	N3—C11—N4	115.86 (18)
C2—C1—H1B	109.5	N3—C11—S2	126.07 (16)
H1A—C1—H1B	109.5	N4—C11—S2	118.07 (16)
C2—C1—H1C	109.5	O2—C12—N4	121.47 (19)
H1A—C1—H1C	109.5	O2—C12—C13	122.65 (18)
H1B—C1—H1C	109.5	N4—C12—C13	115.82 (18)
C16—C2—C1	109.5 (2)	C12—C13—C17	109.11 (18)
C16—C2—C15	111.0 (2)	C12—C13—C18	108.09 (19)
C1—C2—C15	108.7 (2)	C17—C13—C18	109.67 (19)
C16—C2—C3	108.65 (19)	C12—C13—C14	111.20 (17)
C1—C2—C3	110.51 (19)	C17—C13—C14	108.9 (2)
C15—C2—C3	108.50 (18)	C18—C13—C14	109.9 (2)
O1—C3—N1	122.32 (19)	C13—C14—H14A	109.5
O1—C3—C2	121.91 (19)	C13—C14—H14B	109.5
N1—C3—C2	115.75 (18)	H14A—C14—H14B	109.5
N2—C4—N1	115.32 (18)	C13—C14—H14C	109.5
N2—C4—S1	125.71 (16)	H14A—C14—H14C	109.5
N1—C4—S1	118.96 (16)	H14B—C14—H14C	109.5
N2—C5—C10	109.84 (16)	C2—C15—H15A	109.5
N2—C5—C6	109.84 (16)	C2—C15—H15B	109.5
C10—C5—C6	111.57 (17)	H15A—C15—H15B	109.5
N2—C5—H5	108.5	C2—C15—H15C	109.5
C10—C5—H5	108.5	H15A—C15—H15C	109.5
C6—C5—H5	108.5	H15B—C15—H15C	109.5
C7—C6—C5	112.79 (17)	C2—C16—H16A	109.5
C7—C6—H6A	109.0	C2—C16—H16B	109.5
C5—C6—H6A	109.0	H16A—C16—H16B	109.5
C7—C6—H6B	109.0	C2—C16—H16C	109.5
C5—C6—H6B	109.0	H16A—C16—H16C	109.5
H6A—C6—H6B	107.8	H16B—C16—H16C	109.5
C8—C7—C6	110.14 (19)	C13—C17—H17A	109.5
C8—C7—H7A	109.6	C13—C17—H17B	109.5
C6—C7—H7A	109.6	H17A—C17—H17B	109.5
C8—C7—H7B	109.6	C13—C17—H17C	109.5
C6—C7—H7B	109.6	H17A—C17—H17C	109.5
H7A—C7—H7B	108.1	H17B—C17—H17C	109.5
C9—C8—C7	110.28 (19)	C13—C18—H18A	109.5
C9—C8—H8A	109.6	C13—C18—H18B	109.5
C7—C8—H8A	109.6	H18A—C18—H18B	109.5
C9—C8—H8B	109.6	C13—C18—H18C	109.5
C7—C8—H8B	109.6	H18A—C18—H18C	109.5
H8A—C8—H8B	108.1	H18B—C18—H18C	109.5

### Hydrogen-bond geometry (Å, °)

**Table:**

*D*—H···*A*	*D*—H	H···*A*	*D*···*A*	*D*—H···*A*
N2—H2···O1	0.86	1.97	2.650 (2)	135
N3—H3···O2	0.86	1.99	2.670 (2)	135
N1—H1···O2^i^	0.86	2.25	3.078 (2)	161
C1—H1B···O2^i^	0.96	2.54	3.462 (3)	161

Symmetry codes:  (i) −*x*, −*y*, −*z*.
